# ‘There’s a Little Bit of Tension There’: perspectives of mothers and early childhood educators on breast-feeding in child care centers

**DOI:** 10.1017/S1368980024002313

**Published:** 2025-01-07

**Authors:** Jill R Demirci, Rachel Dieterich, Melissa Glasser, Caroline Harpel, Timothy Shope

**Affiliations:** 1 Department of Health Promotion & Development, University of Pittsburgh School of Nursing, Pittsburgh 15261, PA, USA; 2 Chatham University, Nursing Programs, Pittsburgh, PA, USA; 3 Highmark Wholecare, Pittsburgh, PA, USA; 4 Department of Pediatrics, Division of General Academic Pediatrics, University of Pittsburgh School of Medicine, Pittsburgh, PA, USA

**Keywords:** Breast-feeding, Lactation, Child care, Child daycare centres, Workplace, Qualitative

## Abstract

**Objective:**

To explore mothers’ and early childhood (EC) educators’ experiences of breast-feeding/breast milk provision and breast-feeding support in child care centres (CCC) in the USA.

**Design:**

We conducted one-time, semi-structured phone interviews with mothers and EC educators to examine perceptions of support, accommodations and barriers to breast-feeding in CCC. We administered a background survey to assess participant characteristics and quantify perceived degree of breast-feeding support in the workplace (mothers) and CCC (mothers and EC educators).

**Setting:**

US-based CCC

**Participants:**

Fifty working mothers using CCC for their infants and twenty-two EC educators

**Results:**

Interview themes and background surveys reflected neutral feelings towards breast-feeding support received (mothers) and provided (EC educators) in CCC. Maternal expectations for breast-feeding support in CCC were generally low; workplace and social support for breast-feeding were perceived as the most important factors impacting breast-feeding. EC educators’ capacity to offer breast-feeding support was constrained by CCC infant feeding regulations, inadequate breast-feeding training and time limitations. Tensions arose when mothers attempted to manage low milk supply at the CCC level by requesting EC educators to individualise feeding or milk storage practices for their infant.

**Conclusions:**

Breast-feeding efforts of working mothers are undermined in multiple settings, including the workplace and CCC. Improving breast-feeding outcomes for this population requires structural/policy changes that: (1) maximise opportunities for continued, direct breast-feeding and maternal/infant proximity and (2) enforce evidence-based CCC feeding protocols and standards and EC educator lactation training.

The importance of breast-feeding is well established, with dose-dependent health implications for both lactating parents and their breastfed children^(1)^. There is additive benefit to breast-feeding among infants and children attending child care centres (CCC) (i.e. daycare); breast-feeding can prevent or reduce the severity of communicable diseases that are prevalent in CCC^(2–4)^, such as respiratory tract infections^(5)^, gastrointestinal and diarrhoeal illness^(6)^ and otitis media^(7)^. Paradoxically, however, infants in CCC are at elevated risk of breast-feeding discontinuation. A nationally representative cohort study with over 7500 US infants demonstrated that those enrolled in CCC had 1·3 times the risk of discontinuing breast-feeding before 6 months compared with those in parental care^(8)^.

Reduced breast-feeding among children attending CCC is also problematic because of the potential number of families affected. In 2019, centre-based child care was the most common non-relative child care arrangement for children prior to school entry in the USA, with 32 % of children under 1 year cared for in this setting^(9)^. Lack of access to paid parental leave in the USA requires many families to utilise non-family-based child care arrangements upon return to work. The USA is the only industrialised nation without guaranteed paid parental or maternal leave policies^(10)^, which compels many parents to return to work days or weeks following childbirth. Lactating parents who return to work shortly after birth can have difficulty maintaining milk supply and breast-feeding, compared with women who can maintain proximity to their infants and continue to breastfeed on demand. Researchers have found a strong positive association between paid maternity leave length and breast-feeding duration and exclusivity^(11–13)^.

While the impact of paid leave and employer support on breast-feeding is well established^(14,15)^, less is known about the role of CCC in breast-feeding maintenance. There are no legal standards in the USA for breast-feeding support and handling and provision of breast milk in CCC, and substantial variation exists among state-based breast-feeding regulations^(16)^. Both the Surgeon General and the Centers for Disease Control and Prevention (CDC)^(17,18)^ called on US states and territories to implement breast-feeding support in CCC and breast-feeding training for CCC providers based on the standards from the National Resource Center (NRC) for Health and Safety in Child Care and Early Education^(19)^. However, evidence of implementation, monitoring and adherence to these breast-feeding support standards is scant. In a 2022 national analysis, only fifteen states had developed a Breastfeeding Friendly Child Care designation programme designed to recognise CCC meeting some or all of NRC’s guidelines, and these designations often relied solely on CCC self-assessment^(20)^. Our team’s integrative review examining breast-feeding support and practices in CCC describes absent or inconsistently followed breast-feeding policies^(21)^. The purpose of the current study was to explore the experience of breast-feeding/breast milk provision and breast-feeding support in US-based CCC from the perspective of mothers and early childhood (EC) educators.

## Methods

### Recruitment, sample and setting

From April to September 2018, we recruited and interviewed mothers of infants enrolled at CCC and EC educators employed at CCC within the USA. Mothers and EC educators were recruited separately, and their data were therefore not linked. Interviews addressed experiences with breast-feeding and provision of human milk in CCC. Hereafter, unless otherwise specified and to adhere to the terminology used by participants, our use of the term ‘breast-feeding’ refers to any method used to feed an infant their parent’s own milk, including direct chest/breast-feeding and feeding expressed milk via a device like a bottle. We also use the term ‘mother’ and ‘maternal’ here, as our recruitment advertising, eligibility criteria and other study materials used these terms. We acknowledge that not all breast-feeding/lactating parents identify as mothers. This study was approved by the University of Pittsburgh Human Research Protection Office.

Participants were recruited through a national social media advertising campaign through a research recruitment platform (Trialspark). Interviews were conducted after verbal informed consent was obtained. Mothers were eligible if the following criteria were met: (1) ≥ 18 years old, (2) working in a paid position ≥ 15 h per week, (3) mother to an infant 12 months or younger enrolled in a CCC ≥ 15 h per week and (4) breastfed the index infant during the month prior to CCC enrolment. EC educators were eligible if they met the following criteria: (1) ≥ 18 years old, (2) employed full-time in a CCC (≥ 36 h per week) and (3) currently providing care to infants 12 months and younger in the CCC ≥ 20 h per week.

We planned to enrol a maximum of fifty mothers and thirty EC educators. We used maximum variation sampling to purposively recruit participants with variation in characteristics expected to impact experiences with breast-feeding in CCC and most amenable to targeting through advertisements, including geographical areas and underrepresented groups in terms of race/ethnicity, prior/current breast-feeding, CCC type and EC educator years of experience. These are factors associated with breast-feeding uptake, breast-feeding rates within CCC^(21)^ and/or understudied issues that we considered potentially influential in breast-feeding within the CCC environment. As the study progressed, we modified advertisements and participant selection to target those characteristics for which we did not have sufficient representation. Other characteristics known to impact breast-feeding practices, including income level for example, were not included in our selection frame because of potential participant sensitivity to these items, as well as cost limitations in multiple modifications of advertisements through our recruitment platform. Recruitment ceased when we noted significant redundancy in themes. With the rapid recruitment of mothers and wider variations in maternal (as compared with EC educator) experiences, we continued maternal enrolment for approximately ten interviews beyond saturation.

### Data collection

Data collection occurred by phone. After consent was obtained, we administered a background survey assessing demographics and personal breast-feeding experience. For mothers, surveys also included questions on current employment (e.g. position, setting and hours), milk expression and infant feeding practices while working, CCC characteristics, any other child care arrangements for the index infant and a six-item workplace lactation support scorecard modified for brevity and accessible language for the lay public^(22)^ (*α* = 0·293; Table 1). We also administered a fourteen-item five-point Likert scale questionnaire assessing agreement with existence and quality of lactation support at the infant’s CCC (*α* = 0·79; Tables 1 and 2). This questionnaire was adapted for parental relevancy from an eighteen-item dichotomous (yes/no) version of the survey^(23)^.

**Table 1.** Maternal participant characteristics assessed via self-report at time of interview (≤ 12 months postpartum; *n* 50)

Characteristic	Mean	sd
Age (years)	31·5	5·0
Range	18–45	

	***n***	**%**
Race		
White	30	60
Black/African American	4	8
Asian or Indian	7	14
Mixed/biracial and other	9	18
Ethnicity		
Hispanic	7	14
Non-Hispanic	43	86
Marital status		
Married	38	76
Living with a partner	3	6
Single	9	18
Highest level of education		
Associate’s degree or less	10	18
Bachelor’s degree	14	28
Master’s degree or higher	26	54
Number of children		
1	34	68
2 or more	16	32
Age of youngest child		
2–3 months	12	24
4–5 months	11	22
6–7 months	7	14
8–11 months	20	40
Workplace setting		
Office	30	60
School	4	8
Hospital	6	12
Retail or manufacturing	2	4
Other	8	16
Hours worked per week		
10–19	2	4
20–29	6	12
30–34	5	10
35–40	26	52
More than 40	11	22
What is the longest single separation from your baby in a typical week due to work?		
4–8 h	17	34
9–12 h	29	58
13–24 h	3	6
More than 24 h	1	2
How have you fed your youngest child, prior to introducing solid foods?		
Breast milk only	32	64
Breast milk and formula^*^	18	36
Do you express or pump milk while separated from your baby for work?		
No	9	18
Yes, occasionally (not every day)	2	4
Yes, regularly (every day)	39	78
About how many times do you express/pump milk in a usual day while separated from your baby due to work? (*n* 39)		
1–2	24	48
3–4	14	28
5–6	1	2
My work has a written policy on breast-feeding for employees^†^		
Yes	19	38
No	17	34
Unsure	14	28
My workplace provides a private space for employees to express breast milk^†^		
Yes	41	82
No	6	12
Unsure	3	6
My employer provides access to breast pumps at the worksite or through my insurance^†^		
Yes	27	54
No	18	36
Unsure	5	10
My employer provides flexible paid or unpaid break times to allow mothers to pump breast milk^†^		
Yes	45	90
No	4	8
Unsure	1	2
My employer provides free or subsidised breast-feeding support groups or educational classes^†^		
Yes	6	12
No	35	70
Unsure	9	18
My employer offers paid maternity leave, separate from any accrued sick leave, annual leave or vacation time^†^		
Yes	19	38
No	27	54
Unsure	4	8
In your opinion, how supportive of breast-feeding is your place of employment?		
Not at all supportive, not supportive or unsure	3	6
Somewhat supportive	6	12
Supportive	20	40
Very supportive	21	42
Type of child care centre infant attends		
Faith-based	5	10
Community-based or independently owned	29	58
Chain or franchise	12	24
Employer-sponsored or other	4	8
Location of child care centre		
Rural (town or location with < 2500 inhabitants)	6	12
Urban (city)	20	40
Suburban (neighbourhood outside city)	24	48
Is your child care centre accredited by the National Association for the Education of Young Children (NAEYC)?		
Yes	22	44
No	5	10
Unsure	23	46
What is your child care centre’s policy for staff-to-child ratio for infants 12 months or less?		
1 staff for every 3 infants	11	22
1 staff for every 4 infants	26	52
1 staff for every 5 or 6 infants	3	6
Other	6	12
Is your child in a dedicated infant room in your child care centre (i.e. a room for ONLY infants 12 months or younger)?		
Yes	42	84
No	8	16
Does your child care centre have a written policy for storage and handling of breast milk?		
Yes	11	22
No	31	62
Unsure	8	16

* Inclusive of two participants who endorsed feeding some formula in first days or weeks following birth, but then transitioned to exclusively breast milk.

† Adapted items from an employer lactation support scorecard available at: https://www.workwellnc.com/scorecard-maternal_and_lactation_support.php.

**Table 2.** Mother and EC educator ratings for items assessing perceptions of breast-feeding support at their child care centre^*^

ECE centre breast-feeding support category	Item	Mother		EC educator	
		Median rating	IQR	Median rating	IQR
Breast-feeding visits	Mother: Staff at my child’s child care centre encourage me or other mothers to visit and breastfeed during the day EC educator: Our centre/staff encourages mothers to visit and breastfeed during the day	4	2	5	1
Breast-feeding support discussions	Mother: During initial meetings with me, my child’s child care centre discussed how breast-feeding is supported in the facility and by staff EC educator: When meeting with new families, our centre staff discusses how breast-feeding is supported in the facility and by our staff	4	1·5	4	1
Breast-feeding signage	Mother: There is a sign or poster visible in the child care centre so that mothers know breastfed babies are welcome EC educator: There is a sign or poster visible in our centre so that mothers know breastfed babies are welcome	2	1	2·5	2·25
Breast-feeding space	Mother: My child’s centre has a comfortable, private, non-bathroom space available for mothers to nurse their infants before or after work EC educator: Our centre has a comfortable, private, non-bathroom space available for mothers to nurse their infants before or after work	3	2	4	1·25
Breast-feeding support for ECE staff	EC educator: Our centre ensures that mothers employed by the centre have reasonable breaks each day to express milk, and that they have a private space other than a bathroom in which to do so	–		4	2
Unbiased breast-feeding materials	Mother: My child’s centre offers written materials on breast-feeding that are easy to understand and are not produced by formula companies EC educator: Our centre offers written materials on breast-feeding that are easy to understand and are not produced by formula companies	2	1	2	2
EC educator understanding of breast-feeding materials	EC educator: I know and understand the information presented in any available written materials about breast-feeding	–		5	1·25
EC educator knowledge about breast-feeding resources	Mother: Staff at my child’s centre are knowledgeable about community breast-feeding resources like support groups, WIC breast-feeding coordinators and lactation consultants EC educator: I feel prepared to tell mothers about community breast-feeding resources like support groups, WIC breast-feeding coordinators and lactation consultants	2·5	1	2	2
ECE breast-feeding resource contacts	EC educator: Our center has contact information available to refer mothers to community breast-feeding resources as needed	–		3	3
Initial feeding plan	Mother: Staff developed a feeding plan with me when my baby enrolled EC educator: Our centre staff develop an infant feeding plan with each family as infants enrol	4	1	2	1
Feeding plan adjustments	Mother: My child’s feeding plan is updated as my baby grows and moves through the stages of development EC educator: Infant feeding plans at our centre are updated as the infant grows and moves through the stages of development	4·5	1	2	0
Adherence to feeding plan	Mother: My baby would NEVER be given food or drink other than my expressed milk if not indicated in the feeding plan EC educator: Breastfed babies are NEVER given food or drink other than the mother’s expressed milk if not indicated in the feeding plan	5	1	3	0
Feeding style	Mother: My baby is fed by staff based on hunger and fullness cues, rather than a timed schedule EC educator: Infants in our centre are fed based on hunger and fullness cues, rather than timed schedules	4	2	4	1·25
Breast milk storage	Mother: There is refrigerator and freezer space available for pumped breast milk at my child’s centre EC educator: There is refrigerator and freezer space available for pumped breast milk at our centre	5	1	4	0
Breast milk labelling	Mother: Pumped breast milk in my child’s centre must be labelled with the infant’s full name and the date it was pumped for all infants EC educator: Pumped breast milk is labelled with the infant’s full name and the date it was pumped for all infants	4	1	5	1
Back-up breast milk storage	Mother: My child’s centre encourages mothers to provide a small back-up supply of milk if pick-up is delayed or the baby needs more EC educator: Our centre encourages mothers to provide a small back-up supply of milk if pick-up is delayed or the infant needs more	4·	1·25	4	0
EC educator breast-feeding training breadth	Mother: Staff in my child’s centre seemed well trained in the benefits of breast-feeding, how to prepare, feed and store human milk, as well as breast-feeding resources available to families EC educator: Our staff are trained in the benefits of breast-feeding, how to prepare, feed and store human milk, as well as breast-feeding resources available to families	4	1	4	0·5
EC educator breast-feeding training timing	EC educator: Our staff receives breast-feeding training shortly after being hired	–		2	3

EC, early childhood; ECE, early childhood education; IQR, interquartile range; WIC, Special Supplemental Nutrition Program for Women, Infants, and Children.

Missing data were found to be MCAR (missing completely at random). SPSS was used to complete an expectation maximisation (EM) imputation for missing values.

* Items in both the EC educator and mother assessment adapted from Garth, E., A.L. Messer and D.L. Spatz, Child Care Centers’ Role in Support of Breastfeeding Families. MCN Am J Matern Child Nurs, 2016. 41(3): p. 154-61. Items answer choices were on a Likert scale: 1 = Strongly Disagree; 2 = Disagree, 3 = Neutral/Unsure, 4 = Agree and 5 = Strongly Agree. Cronbach’s *α* for EC educator (18 items): 0·619. Cronbach’s *α* for mothers (14 items): 0·791.

Surveys for EC educators addressed past and current employment experience in child care, characteristics of participants’ CCC (Table 3) and an eighteen-item Likert scale questionnaire assessing agreement with existence and quality of lactation support at participants’ CCC (*α*: 0·619). Likert scale items were adapted from a dichotomous (yes/no) version of the questionnaire to capture nuance in implementation of lactation supports (Table 2)^(23)^.

**Table 3.** EC educator characteristics assessed via self-report at time of interview (*n* 22)

Characteristic	Mean	sd
Age (years)	30·4	6·0
Range	20–42	

	***n***	**%**
Race		
White/Caucasian	19	87
Black/African American	3	13
Ethnicity		
Hispanic	1	5
Non-Hispanic	21	95
Marital status		
Married	13	59
Living with a partner	1	5
Single	8	36
Highest level of education		
Some college, no degree	6	27
Associate’s degree	5	23
Bachelor’s degree or higher	11	50
Biological children	15	68
How did you feed your children when they were infants, prior to introducing solid foods? (*n* 15, participants with children)		
Breast milk only	5	33
Formula only	1	7
Breast milk and formula^*^	9	60
Years employed in child care or education		
1–3	6	27
4–6	6	27
7 or more	12	46
Location of child care centre		
Rural	5	23
Suburban	9	41
Urban	8	36
Type of child care centre		
Faith-based	5	23
Community-based/independently owned	12	55
Chain or employer-based	5	22
Is your child care centre accredited by the National Association for the Education of Young Children (NAEYC)?		
Yes	9	41
No	12	55
Unsure	1	5
Do you work in a dedicated infant room in your child care centre (i.e. room for ONLY infants 12 months or younger)	14	64
What is your child care centre’s policy for staff-to-child ratio for infants 12 months or less?		
1 staff for every 3 infants	4	18
1 staff for every 4 infants	13	59
1 staff for every 5 infants	3	14
Other^†^	2	9
Estimated number of infants under 6 months of age you care for at your child care centre on a typical day	4·1	2·8
Range	1–12	
Estimated number of infants between 6 and 12 months of age you care for at your child care centre on a typical day	3·8	3·2
Range	1–12	
Does your daycare centre have written policy for breast milk storage and handling?		
Yes	19	87
No/unsure	3	13
How would you rate your knowledge of the DETAILS of the written policy for storage and handling of breast milk in your child care centre? (*n* 21)		
Very good	13	62
Good	7	33
Moderate	1	5
Poor	0	0
Very poor	0	0

EC, early childhood.

* Inclusive of one participant who fed only formula for first three children and then only breast milk for her fourth child.

† One participant reported two staff for every five infants; one participant reported three staff for every ten children under age of 2 years.

Following surveys, JRD or MG, both trained in qualitative interviewing, conducted audio-recorded interviews, which were professionally transcribed. Interviews followed a semi-structured interview guide, modified as the study progressed to establish convergence and divergence in themes. The maternal interview guide assessed experiences and decision-making around breast-feeding in the context of both employment and having an infant regularly attend a CCC. For EC educators, the interview guide assessed supports and barriers for breast-feeding families at the CCC, personal feelings on breast-feeding, centre breast-feeding training and the CCC’s infant feeding regulations and processes. Both groups’ interview guides included questions about desired improvements lactation support in CCC and beyond. Participants were compensated $25.

### Analysis

We used SPSS v. 28 to calculate summary statistics for survey data. RV and CH trained in qualitative analysis independently coded mother and EC educator interviews, respectively, following codebook development. The codebook was created through discussion and review of five maternal interviews with MG, JRD and RV and later expanded and refined for EC educator transcripts with JRD and CH. Interviews were coded with conceptual labels using qualitative analysis techniques described by Corbin and Strauss^(24)^ and ATLAS.ti software^(25)^. Codes were iteratively collapsed, expanded, defined and refined by coders as analysis proceeded and developed into categories and interconnected themes. Approximately 25 % of interviews (*n* 10 mother interviews, *n* 7 EC educator interviews) were double-coded by author MG to ensure consistent application of codes. All interviewers and analysts were White women of childbearing age – all but one without experience as a parent using a CCC. We used several techniques to aid analysis, including individual interview summaries, interview ‘titles’ to capture the most salient categories/theme(s) and matrices to compare interviews on participant characteristics and major code categories/themes^(26)^.

## Results

**Fig. 1:**
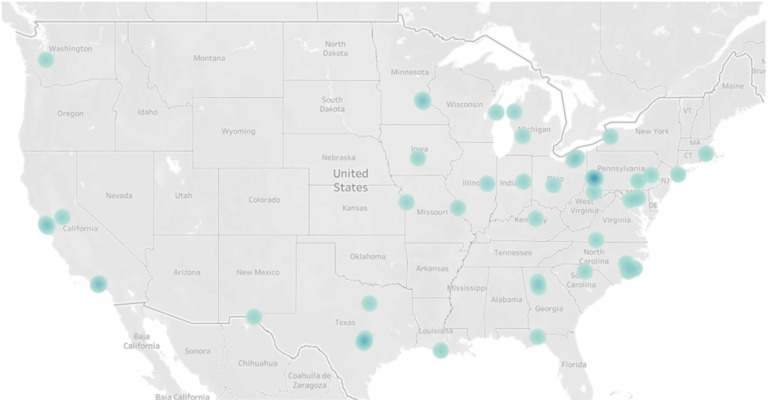
Geographical representation of maternal participants (*n* 50; blue dots), with darker colouring representing zip codes with higher concentration of participants.

**Fig. 2:**
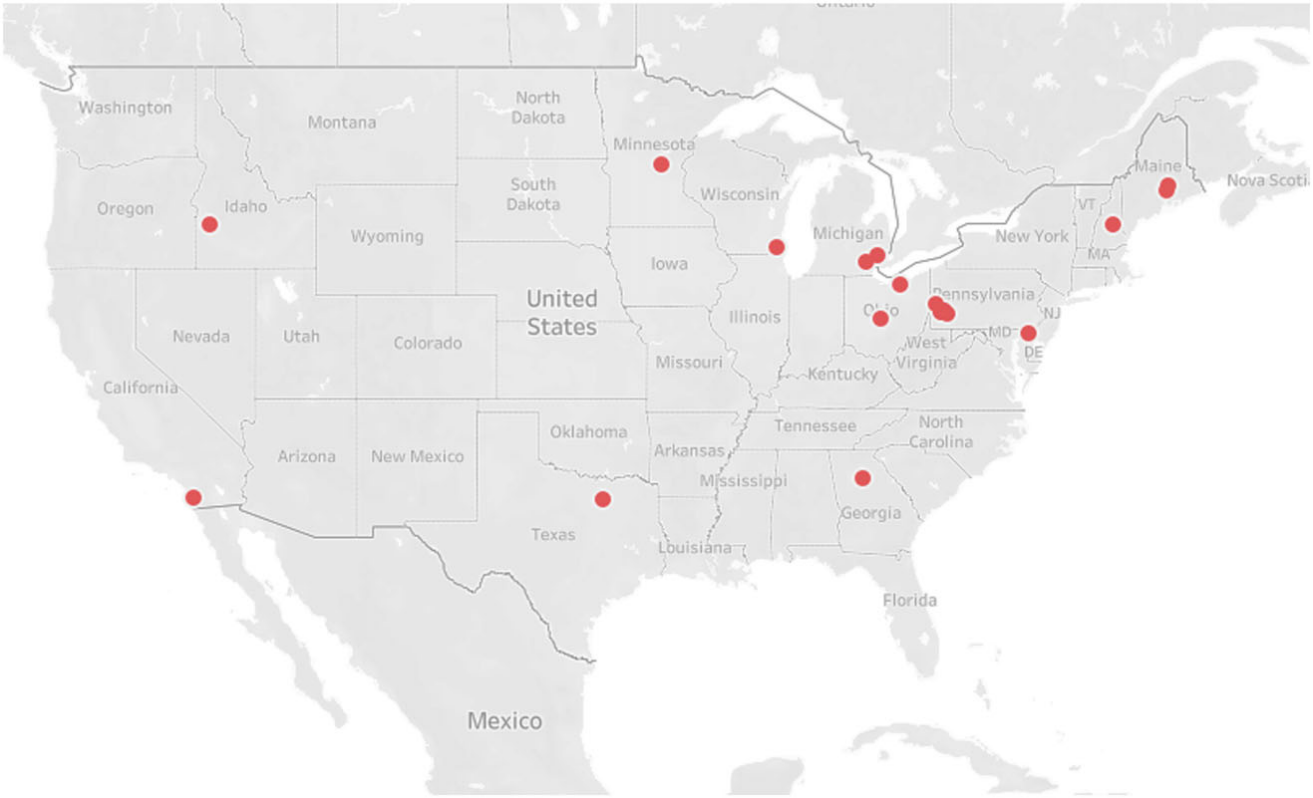
Geographical representation of EC educator participants (*n* 22; red dots). EC, early childhood.

We interviewed fifty mothers and twenty-two EC educators. In both groups, participants were majority non-Hispanic White, married and held a Bachelor’s degree or higher (Tables 1 and 3). Maternal participants were from twenty-three different states and concentrated in the northeast and Midwest (Fig. 1). EC educators were from twelve different states and overrepresented in the northeast and upper Midwest (Fig. 2).

Most mothers worked in an office, worked ≥ 35 h per week, expressed milk at work at least once or twice per d and felt their workplace was ‘supportive’ or ‘very supportive’ of breast-feeding, though > 60 % (*n* 31) reported not having a paid maternity leave. Most mothers disagreed that there was a written policy on storage and handling of breast milk at their CCC but agreed that their centre upheld most other assessed breast-feeding support measures, including use of feeding plans and having EC staff who were well trained to prepare and feed human milk (Table 2).

Most EC educators had ≥ 4 years experience in child care, had biological children and combination-fed their children (formula and breast-feeding) as infants. Most also agreed or strongly agreed that their centre upheld ten of the eighteen assessed breast-feeding support indicators (Table 2).

### Qualitative findings: maternal participants

Collectively, maternal participants expressed neutral feelings about their breast-feeding experiences in CCC, though some voiced more positive or negative encounters. Workplace barriers to breast-feeding were more prominent than CCC barriers. Four themes summarised the breast-feeding experiences of mothers.

#### Worth the work

Participants were determined to breastfeed ‘no matter what’, because they felt it was best, particularly because their infant attended a CCC. Perceived benefits and reasons for maintaining breast-feeding while back to work and using CCC, despite its challenges, included child immunity and health benefits, bonding and the economic burden of formula.

*My baby is sick a lot because she’s in daycare. And I get sick a lot because she’s in daycare. And knowing that there are antibodies in the breastmilk that might help her when she’s sick has also made me want to continue at least until she’s a year old.*

Participants who introduced formula or weaned earlier than they intended often did so due to significant challenges with maintaining a sufficient milk supply. Formula use was perceived as matter of need and convenience.

#### They don’t care what’s in the bottles

Participants found that CCC would support ‘whatever the parents want to do [with infant feeding]’ within the confines of policy, though EC educators rarely went ‘above and beyond’ to support breast-feeding dyads (with some exceptions). However, participants generally did not perceive this as problematic and had minimal expectations for breast-feeding support at CCC.

*They didn’t have things for me to read or look at about breastfeeding. It was more, I knew beforehand that this was what I wanted to do, and essentially, as long as I followed their protocol, then it was fine.*

*Basically, they [EC educators] have us make bottles. They don’t care what’s in the bottles. They’re like, ‘make the bottles, put them in the fridge, we will feed them to your baby’. …the way they set it up it wouldn’t matter if it’s breast milk or formula. And they don’t encourage me to come in [to breastfeed].*

Participants felt EC educators lacked knowledge about breast milk feeding, handling and storage and described educating EC staff on these topics. In some cases, participants were the only families at their CCC providing breast milk, which was cited as a possible reason for low breast-feeding knowledge among EC educators. Several participants experienced anxiety in sending their child to a CCC whose providers had not cared for breastfed infants. In one instance, this led a participant to transition from breast milk to infant formula for CCC feedings.

#### A lot of pressure

Stress around infant feeding primarily existed at the intersection of work demands that made regular milk expression difficult (resulting in low milk volume) and CCC infant feeding practices that ‘wasted milk’ or made it difficult to ‘keep up’ adequate milk production. Tensions arose around human milk storage and disposal requirements at the CCC which necessitated the mother bringing in more milk than the infant consumed, as well as feeding methods that were perceived to lead to infant overconsumption. For example, mothers sometimes met resistance from CCC when they requested cue-based/on-demand feedings or paced bottle-feeding – practices that required more EC educator time but were considered more responsive feeding methods that could conserve milk. Some participants perceived that EC staff were ‘happier’ when infants were ‘overfed and sleepy’.

*When we have family watching him, we start the bottles a little bit smaller and then ask them to add milk as needed so we don’t waste any. Where[as] at daycare, we have to anticipate ‘this is the most he could possibly eat’, and then some gets wasted. So that’s a challenge at daycare.*

*I’d be like, ‘well, where is it [breast milk]? Can I have it? Can I take it home? Could you give it to him tomorrow?’ And they [EC educators] would have dumped it out. And I know that there are state handling guidelines and whatnot that they abide by. But…you put a lot of pressure – I’m not an overproducer by any stretch…so yeah, it does cause me anxiety when I hear they dump any out.*

Participants experienced other sources of breast-feeding-related stress at CCC. The labour involved in expressing milk, cleaning bottles and pump equipment, and preparing labelled bottles of expressed milk daily for EC staff was described as tedious, ‘like a second job’, and ‘not sustainable’. Some participants described CCC without designated breast-feeding spaces and discouragement of unscheduled drop-ins for breast-feeding – ‘[EC educators] don’t really want you to come…and then leave…because it gets the kids all flustered’. Coming into CCC to breastfeed during the workday was also difficult because of the time required to travel back and forth to work and that infants became distracted while nursing and ‘clingy’ after breast-feeding when they needed to return to work.

#### Support is key

Participants described the importance that strong social support systems played in their ability and desire to maintain breast-feeding upon returning to the workplace. Partners provided substantial logistic, emotional and moral support for participants, including assistance with household chores, preparing bottles with expressed milk for the EC staff and encouragement to ‘keep going’.

Economic privilege was critical in participants’ capacity to continue to express milk and breastfeed. Those with financial means were able to purchase quality breast pumps and accessories, multiple pumps for different settings (e.g. home and work) and were often able to delay return to the workplace longer to establish a robust milk supply.

Workplace breastfeeding support, both in terms of policy and culture (e.g. ‘a pro breast-feeding climate’), was viewed as the most important factor determining participants’ ability to maintain breast-feeding. Access to paid, extended leave was viewed as critical. Participants felt supported to breastfeed in the workplace when they had paid breaks for pumping and/or visits to the CCC to breastfeed, flexible work hours, health insurance benefits that provided quality electric breast pumps and private lactation rooms at work that could accommodate more than one breast-feeding/pumping parent. Across employment settings, lack of accommodations (e.g. time and space) to express milk at work led to problems keeping up adequate milk supply. Several participants noted that onsite child care at work had the potential to solve most of their struggles with maintaining breast-feeding upon return to employment:

*In a dream world, daycare would be right here at work, and I could just walk next door and feed him and come back. I think that would be so much easier.*

### EC educators

EC educators wanted to support breast-feeding parents and found ways to do so. However, they acknowledged conflict between parental feeding expectations, their own lack of breast-feeding knowledge and training, and seemingly arbitrary CCC regulations for breast milk handling. Two themes captured EC educators’ experiences.

#### We get it

Collectively, participants described a supportive attitude towards breast-feeding at their CCC (‘we will do whatever we can to help you’) and noted health and bonding benefits of breast-feeding. However, they also stressed that they and their colleagues did not provide ‘judgement one way or another for breast milk or formula’ and did not possess strong ideology around infant feeding. Some mentioned breast milk feeds were ‘easier’ and less time-consuming than formula feeds, because breast milk does ‘not clump up’, does not need reconstituting and is brought to the CCC in prepared bottles. Conversely, some participants noted formula preparation was easier, more readily available than breast milk and kept infants satiated and content longer. Some disclosed discomfort in handling breast milk (‘it’s somebody else’s bodily fluids…it takes a little bit getting used to getting the milk spit up all over you’) or seeing parents breastfeed at the CCC. Participants who expressed discomfort included those who did not have children and those who had fed their children both infant formula and their own milk.

EC educators’ personal breast-feeding experiences engendered a sense of solidarity with breast-feeding parents, such that they felt comfortable offering advice, support and ‘going against a couple of the silly rules’ regarding milk handling (e.g. not wearing gloves to warm milk, saving bottles of leftover breast milk in the refrigerator for the parent to take home). EC educators with personal breast-feeding experience also described educating other staff on breast-feeding.

*I would say that the biggest determining factor [for how I support breastfeeding parents] was when I had my own son… because of some difficulties that we had…I really did like, a lot of research and was in a couple of support groups…I was able to bring that new information into our child care setting to the benefit, I really feel, of the parents… and also to be able to train staff… Several of [my EC educator colleagues] also breastfed their babies, so they understand, and went back to work, so they/we get it: the whole nursing mom, working thing.*

Regardless of personal breast-feeding experiences, participants understood the challenges and stress mothers experienced with milk expression in the workplace and the ‘pressure’ to keep up their milk supply. They described multiple ways they attempted to ease this burden, including suggestions for parents to make smaller volume bottles to match infant intake, keeping parents updated on their infant’s feeding patterns and encouraging parents to come into the CCC to breastfeed. Some EC educators went further – referring parents to lactation experts, providing research articles on breast-feeding and washing empty bottles. Participants also made special accommodations to try to ease the transition of a breastfed infant into the CCC, including feeding away from other children and distractions, wearing an article of clothing with the mother’s scent, having the infant’s ‘preferred’ EC educator do feedings and recommending different bottle nipple types to parents. These supportive practices did not differ meaningfully based on EC educator personal breast-feeding experiences.

#### We have to do what they (parents, child care centre policymakers/regulators) want

Participants found themselves at the centre of competing demands to support parents’ breast-feeding goals while upholding state regulations and CCC policies for handling of breast milk. This tenuous position was further complicated by a consistent and recognised lack of breast-feeding training of EC educators by CCC. Participants voiced a strong interest in obtaining more education and training about breast milk handling and feeding practices.

*I think we can kind of be considered maybe not as knowledgeable or supportive [as we should be]… We try our best…it’s not like a formal training like we should be doing.*

State regulations and centre policies for handling and storage of breast milk as reported by participants varied widely. Most participants noted regulations required them to discard breast milk or place it back in the child’s cubby for the parent to dispose of after it had been unrefrigerated anywhere from 45 min to 2 h. Some participants noted that their centre allowed them to re-refrigerate leftover breast milk for the parent to decide what to do with it. One participant described a mother bringing in a bag of dry ice for her child’s cubby, so unused milk could be saved and still in compliance with CCC policy not to re-refrigerate. Participants voiced a sense of moral failing, anger, frustration and sadness about having to discard unused breast milk and wasting mothers’ ‘hard work’.

*My one thing is I don’t like dumping it [pumped breast milk] out because I struggled so much with [pumping myself]… And I think if I was home and it was my own milk, I’d stick it back in the fridge.*

Participants’ frustration with CCC human milk storage and feeding policies was often matched by their frustration with breast-feeding parents’ ‘unrealistic’ expectations for specific feeding schedules or volumes. Providers discussed a tension between having ‘to do what [the parents] want’ to conserve their available breast milk and avoid infant formula, while also attending to the baby’s hunger cues. At times, providers deviated from parental-preferred feeding schedules in responding to infant hunger cues.

*[The baby] starts looking hungry before the time comes that her mom wants us to feed her. I tend to, like, try and distract her for a little bit, but I can’t bring myself to not feed a hungry baby. So we’re supposed to be sticking to a schedule… I’m more concerned with feeding the baby than with making her mother happy about her schedule. So there’s a little bit of tension there.*

*My breastfed mommies can walk around at home and feed that baby whenever it cries for a couple of minutes, just a couple sips. We can’t do that [because of caring for other infants and policies for milk disposal]. I need the baby to eat at least a bottle to be happy, v. I can’t feed a baby every half hour.*

## Discussion

Bidirectional tension existed between mothers and EC educators in relation to breast-feeding support in CCC. While the most immediate perceived threat to mothers’ breast-feeding aspirations was insufficient milk supply stemming from lack of workplace lactation accommodations, mothers attempted to manage this issue by asking CCC to conserve expressed milk through measures like adapting their milk storage practices. Mothers expressed frustration when they met resistance on feeding accommodations from CCC and staff. Equally, EC educators felt unable to support breast-feeding mothers and their infants in the ways they wanted due to restrictive infant feeding regulations, insufficient breast-feeding knowledge and training and difficulties inherent in matching a parent’s feeding style while simultaneously maintaining a high level of care for other infants in their charge.

**Fig. 3:**
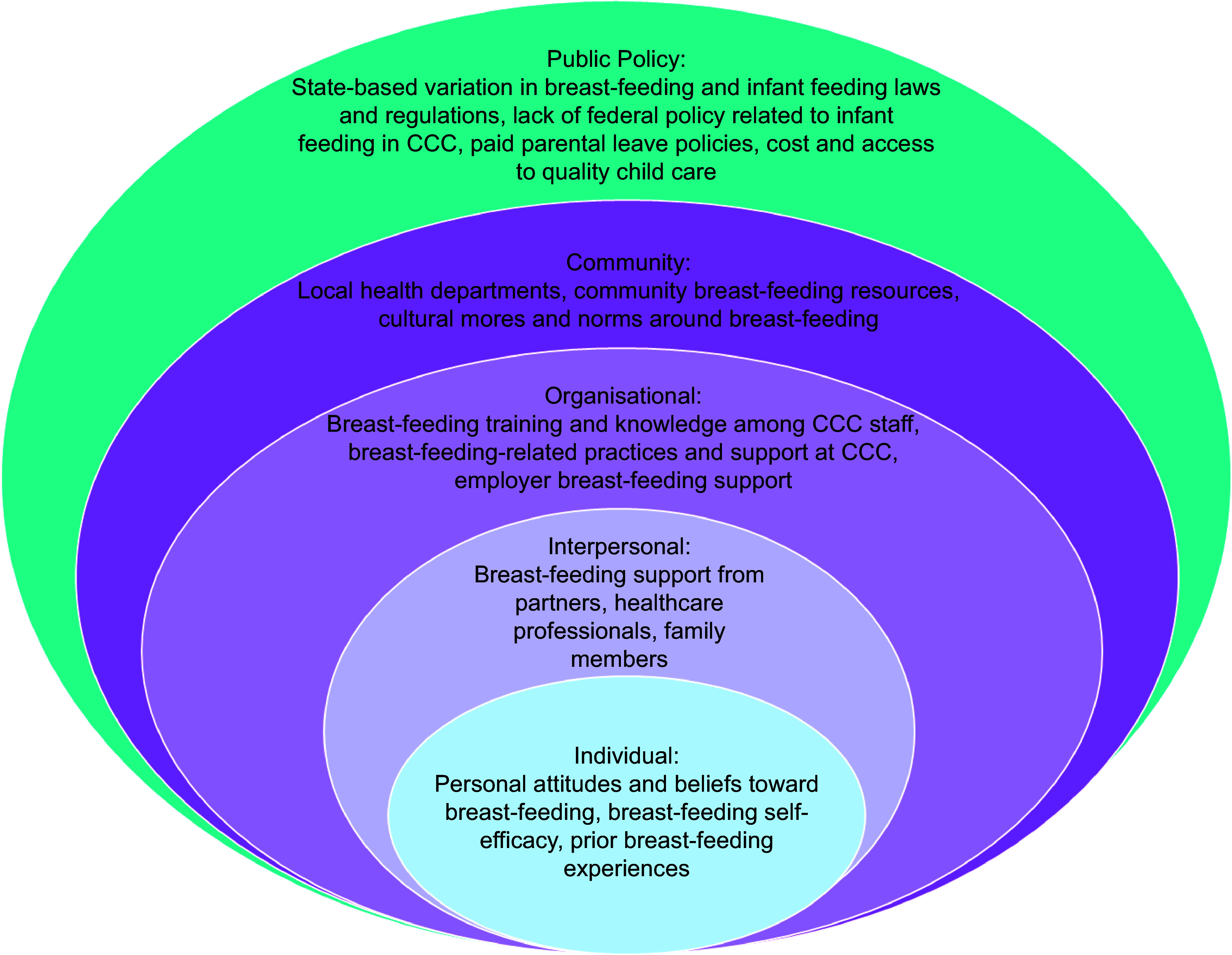
Social Ecological Model conceptualisation of levels of influence on breast-feeding in CCC, as identified by participants and documented in the literature. CCC, child care centres. Note: Author-created rendering/conceptualisation of influences on breast-feeding in CCC based on the Social Ecological Model.

The Social Ecological Model, which conceptualises health and health behaviours as influenced by embedded layers of individual, interpersonal, organisational, community and public policy factors, provides a useful framework to contextualise these findings (Fig. 3)^(27)^. Mothers and EC educators were most attuned to individual and interpersonal (and sometimes organisational) level interactions and actions that impacted their breast-feeding experiences in CCC. Maternal and EC educator participants who had more positive breast-feeding experiences were those who maintained good bidirectional communication about the infant’s feeding at the CCC. Mothers experienced less pressure when their workplace was able to accommodate their pumping needs and schedule. Mothers who are returning to work and planning to use CCC might therefore be counselled, even during pregnancy or early postpartum, to begin conversations with their workplaces and potential CCC about breast-feeding accommodations. Parents may also choose to explore newer technologies, like wearable pumps, that allow pumping to occur discreetly whilst continuing to work and have face-to-face workplace interactions. Likewise, CCC can consider implementing communication systems with parents that prioritise frequent updates or dialogue about evolving infant feeding patterns.

Societal and public policy factors that came to bear on participants’ individual experiences were more rarely discussed (e.g. expansion of CCC workforce, centre quality) – perhaps because their ripple effects are difficult to observe directly or because they are considered immutable. One issue that surfaced in maternal and EC educator interviews spanning the community, organisational and public policy levels of influence was the wide variation in CCC infant feeding procedures, which included human milk storage and preparation. Achieving consensus on breast-feeding guidelines for CCC may initially be most feasible at community and organisational levels, where advocates can work through local health departments, child care resource and referral agencies, and national child care corporations. Consensus guidelines can be modelled from principles of Breastfeeding Friendly Child Care Centers, include having a written breast-feeding policy and training all child care staff on the policy and in the protection, promotion and support of breast-feeding^(28)^. Currently, these elements are consistently absent in US-based CCC^(20,29)^.

At the macro policy level, studies have found substantial state-based variation in breast-feeding and infant feeding related laws and regulations^(16,30)^. While CCC are required to follow state and federal regulations, infant feeding policy change at these levels is complicated by bureaucracy. Absent state and federal policy, however, CCC typically adopt child care recommendations from national organisations. Therefore, focusing efforts on ensuring consistency, clarity and regular evidence-based updates to policy and position statements from such organizations, like the CDC, Head Start, and the American Academy of Pediatrics/Caring for our Children, is a worthwhile endeavour. For milk storage and preparation, for example, CDC guidelines recommend that breast milk leftover from a previous feeding must be used or discarded within 2 h^(31)^, but further details that encompass the range of refeeding scenarios that might occur in a CCC are not elucidated (e.g. initial milk storage conditions). This lack of guidance reflects the absence of rigorous research on the safety and quality of human milk under various storage conditions.

Similar to our findings, other researchers have found limited breast-feeding training and knowledge among child care staff in the USA^(32)^. Although we found few instances of negative attitudes towards breast-feeding or handling/preparation of human milk among EC educators (in contrast to findings of at least one study^(33)^), ambivalent attitudes towards breast-feeding were common. However, EC educators with first-hand positive breast-feeding experience was a pivotal factor in EC educators becoming breast-feeding advocates for parents. The importance of EC educator personal breast-feeding experience is corroborated by a qualitative study of forty-six CCC in Washington State^(34)^. Personal breast-feeding experience of EC educators notwithstanding, national- and state-level adoption of Breastfeeding Friendly Child Care policies have the potential to counteract breast-feeding ambivalence and infant formula feeding norms present in many US CCC^(35)^.

While 82 % of maternal participants indicated that their workplace was ‘supportive’ or ‘very supportive’ of breast-feeding, our qualitative findings highlight the difficulties mothers still experienced combining breast-feeding with return to work, particularly with regard to the pressure to pump large volumes of milk while separated from one’s infant. This underscores the importance of continued attention to accommodations for parents within the labour force, including universal access to extended, paid parental leave, flexible work models, adequate time and space for milk expression or direct breast-feeding, and support for breast-feeding/milk expression from colleagues and supervisors^(14,36–38)^. Our findings also indicate enthusiasm for creative arrangements that would enable parents to more fluidly combine breast-feeding and work, such as onsite workplace child care.

Likewise, there is an urgent need to address inadequate child care availability and quality in the USA, which was exacerbated by the COVID-19 pandemic. While number of CCC and child care employment has returned to pre-pandemic levels, child care workers remain among the lowest paid professionals in the USA, with wages 60 % below the national average in 2023^(39,40)^. In July 2023, the Biden administration took steps to cap out-of-pocket expenses for families using child care and to increase the reliability of payments to child care providers through the Child Care & Development Block Grant (CCDBG) as part of the American Rescue Program^(34)^. Policies like these have potential to strengthen the child care workforce, thereby increasing CCC’s capacity to provide lactation training and lactation support for families.

Our findings have potential implications beyond the USA. While high-income countries that provide paid parental leave frequently also provide high-quality, subsidised child care^(41)^, breast-feeding support may still be lacking. For example, limited breast-feeding training and knowledge among CCC staff was also an identified barrier for breast-feeding support among sixty-two CCC in Adelaide Australia (where child care is subsidised^(42)^) in 2013^(43)^; survey responses indicated that over 60 % centres had no formal or informal breast-feeding training for staff. More recent research on breast-feeding support in child care settings outside the USA, and particularly in low- and middle-income countries, is lacking.

The primary limitation of this study was selection bias, attributable in part to our eligibility criteria and online recruitment strategy, which may have been less likely to reach underserved populations. For example, we did not include non-English speakers, and the high maternal education level indicates probable low representation of low-income mothers. In addition, almost all EC educators were White, whereas nationally, the early childhood education (ECE) workforce is 63 % White and 17 % non-Hispanic Black^(44)^. More than 40 % of maternal and EC educators affirmed that their centre was accredited by the National Association for the Education of Young Children (NAEYC; a marker of high-quality ECE), compared with the national NAEYC accreditation rate of < 12 %^(45)^. Poor representation from racial/ethnic minorities, socio-economically vulnerable groups and under-resourced areas limit generalisability of our findings to families and CCC programmes with lower rates of breast-feeding. EC educator experiences, attitudes and support for breast-feeding and CCC breast-feeding resources, policies and practices may be quite different in these populations. In addition, our eligibility criteria specified that maternal participants must have been employed and provided breast milk in the month prior to CCC enrolment. Thus, we did reach those who stopped breast-feeding or working because of the combined challenge of these activities.

Another limitation was the timing of data collection, which occurred prior to the COVID-19 pandemic. Therefore, we did not capture the seismic shifts that occurred in child care settings during the pandemic, including closures, short-staffing and shifting work environments for many working parents (there were less notable impacts on overall breast-feeding rates as a result of the pandemic^(46–48)^). Some pandemic-related changes have lingered or even been exacerbated after the US Department of Health and Human Services lifted the federal Public Health Emergency in May 2023. A February 2024 report by NAEYC which surveyed over 10 000 EC educators across the USA found that many ECE programmes are facing rising operating costs, recurrent staff shortages and threatened closure after the American Rescue Program child care funding expired in September 2023 and as some parents have shifted to less consistent use of CCC^(49,50)^. With these compounding challenges, it is plausible that breast-feeding support in CCC has been deprioritised and worsened since our data were collected.

### Conclusion

Both mothers and EC educators recognised shortcomings in breast-feeding support at CCC, including in policies for milk storage and feeding and inadequate breast-feeding training of EC educators. These issues, along with maternal workplace factors and the mother’s support system, impacted the duration and quality of mothers’ breast-feeding experiences. Our findings support the need for further research and more detailed guidelines on the safety and nutritional quality of raw human milk under various conditions of storage and feeding/refeeding. In addition, as the US works to expand and increase the quality of the child care workforce, it will be important to prioritise breast-feeding education for EC educators and include breast-feeding rates in CCC as a key marker of quality. Repeating this study with federally funded CCC programmes would provide a sample more reflective of the demographic-heterogeneity within the USA.
